# Caffeine and modafinil counteract sleep deprivation through distinct neurocognitive pathways: an ERP study of object working memory

**DOI:** 10.3389/fnhum.2026.1832731

**Published:** 2026-06-22

**Authors:** Yongcong Shao, Lin Xu, Xin An, Jingwei Zhang, Hongyang Xie, Jingjing Gong, Haishui Duan, Yonghua Huang

**Affiliations:** 1 School of Psychology, Beijing Sport University, Beijing, China; 2 Laboratory of Sports Stress and Adaptation of General Administration of Sport, Beijing, China; 3 The Seventh Medical Center, Chinese PLA General Hospital, Beijing, China; 4 Three Gorges Hospital Affiliated to Chongqing University, Chongqing, China

**Keywords:** 36-hour sleep deprivation, caffeine, event-related potential, modafinil, object working memory

## Abstract

**Background:**

Sleep deprivation impairs core cognitive functions, such as working memory. Previous studies focused on global working memory performance, and direct comparisons of how different cognitive enhancers mitigate sleep deprivation-induced deficits in object working memory remain limited, particularly regarding the underlying neurophysiological mechanisms. This study compared the counteractive effects of caffeine and modafinil after 36 h of total sleep deprivation and elucidated their stage-specific neural mechanisms during object working memory processing.

**Methods:**

A randomized, double-blind, crossover design was used with 14 healthy male participants. Participants completed a 2-back object working memory task at baseline and after 36 h of sleep deprivation under caffeine and modafinil conditions. Event-related potentials were recorded to assess key component changes, including P2, N2, P3, and LPC.

**Results:**

Behaviorally, modafinil significantly improved accuracy after sleep deprivation, surpassing baseline performance, with an exploratory trend toward shorter reaction times. In contrast, caffeine maintained behavioral performance at baseline levels without significant change. Neurophysiologically, caffeine markedly increased P2 amplitude but significantly decreased LPC amplitude after sleep deprivation, reflecting enhanced early perceptual processing alongside a decline in late-stage cognitive maintenance resources. However, modafinil stabilized P2 and LPC responses, suggesting improved neural efficiency and sustained top-down cognitive control during stimulus evaluation.

**Conclusion:**

Although caffeine and modafinil mitigate sleep deprivation induced decline in object working memory, they operate through distinct neural mechanisms. Caffeine relies on generalized compensatory arousal mechanisms, whereas modafinil exerts a more efficient and targeted enhancement of executive control. These findings provide electrophysiological evidence supporting the differential application of cognitive enhancers under extreme sleep loss.

## 1 Introduction

Sleep’s significance for human health is indisputable. However, in the context of rapid societal development and an increasingly fast-paced lifestyle, pressures from heavy academic workloads and job responsibilities have compelled many individuals to reduce sleep duration and allocate more time to work. Sleep deprivation is a growing social issue that is particularly pronounced in specific occupational groups, such as shift nurses, factory workers, drivers, and military personnel, where chronic disruption of circadian rhythms exacerbates sleep deprivation (Craven et al., 2022). Previous studies found that insufficient sleep not only induces negative emotional states, such as tension, irritability, and depression (Palmer et al., 2024), but also undermines the health of various physiological systems, including the cardiovascular, nervous, immune, and endocrine systems (Liu and Chen, 2019; Olsson et al., 2018; Sang et al., 2023). Moreover, sleep deprivation detrimentally affects cognitive function (Khan and Al-Jahdali, 2023; Killgore, 2010), affecting various cognitive domains, including memory (Ashton et al., 2020; Zimmerman et al., 2024), attention (Whitney et al., 2019), alertness (Lim and Dinges, 2008), and decision-making abilities (Killgore et al., 2007). These cognitive impairments increase the likelihood of major risk events, such as workplace accidents and traffic incidents. Consequently, the effective mitigation of cognitive deficits associated with sleep deprivation has become a critical research topic in the fields of psychology and neuroscience.

Humans have long explored various methods to enhance their cognitive abilities (Racine et al., 2021). Additionally, the use of psychoactive substances, particularly those with stimulatory effects, has garnered considerable scientific attention. These substances include readily available stimulants, such as caffeine, and prescription medications, such as modafinil, which are commonly used by healthy individuals to improve alertness and cognitive performance. Considering the cognitive deficits associated with sleep deprivation, the development of effective intervention strategies has become particularly urgent. The prudent application of cognitive enhancers represents a promising strategy for mitigating these impairments.

Caffeine, the most widely consumed cognitive enhancer, is integrated into the daily life of many individuals. Many individuals depend on caffeinated beverages, such as tea or coffee, to maintain cognitive efficiency in academic or occupational tasks, often leading to behavioral dependence. The cessation of caffeine intake may result in fatigue and diminished performance. Caffeine is classified as a xanthine alkaloid and is naturally found in plants, such as coffee, tea, and guarana. As a central nervous system stimulant, it effectively dispels drowsiness, enhances alertness, and enhances reaction time. Consequently, it has been widely employed in cognitive tasks that require sustained attention (Cappelletti et al., 2015; McLellan et al., 2016). Given the global consumption of coffee, tea, soft drinks, and energy beverages, caffeine has become one of the most extensively used psychoactive substances; however, its use is associated with the potential risk of dependence (Franke et al., 2012).

Modafinil is another cognitive-enhancing drug that is increasingly utilized in occupational contexts. This medication was initially approved for the treatment of sleep disorders related to shift work, obstructive sleep apnea, restless legs syndrome, and narcolepsy, which can result in excessive daytime sleepiness (Murillo-Rodríguez et al., 2018). However, its psychoactive and cognition-enhancing effects demonstrated therapeutic potential in other pathological conditions, such as fatigue, somnolence, and cognitive impairment (Sousa and Dinis-Oliveira, 2020). Among healthy individuals, modafinil is used off-label to counteract sleepiness and augment attentional capacity and cognitive performance (Battleday and Brem, 2015). Previous studies indicated that modafinil, a non-amphetaminergic central stimulant (Sousa and Dinis-Oliveira, 2020), exerts notable effects by enhancing alertness and various cognitive functions (Cao et al., 2019; Repantis et al., 2010).

Among various cognitive functions, working memory is a core cognitive process responsible for the temporary storage and online manipulation of information, serving as the foundation for multiple higher-order cognitive functions (Baddeley, 1992). Working memory enables individuals to maintain and manipulate task-relevant information within a limited time frame, thereby playing a critical role in perception, decision-making, and behavioral control (D'Esposito and Postle, 2015). However, working memory is vulnerable to the adverse effects of sleep deprivation. Total sleep deprivation can lead to cognitive resource depletion, resulting in a decline in working memory capacity, as evidenced by prolonged reaction times and decreased accuracy in behavioral tasks (Li et al., 2024).

Sleep deprivation impairs working memory through various neurophysiological mechanisms. It may weaken the activation levels of the frontoparietal control network (Ma et al., 2015), thereby affecting the processes involved in information retention and manipulation within working memory. Additionally, it can reduce the speed of information processing, consequently reducing the efficiency of working memory operations (Casement et al., 2006; Zhang et al., 2019). Previous studies indicated that both total sleep deprivation and partial sleep restriction adversely affect working memory (Hennecke et al., 2021). Although previous studies established sleep deprivation’s detrimental impact on the overall working memory function, differences may exist in the sensitivity of distinct working memory subsystems to sleep deprivation.

Object working memory, a subsystem responsible for processing and maintaining information regarding object characteristics in the visuospatial domain, plays an important role in environmental navigation, object recognition, and interactive behavior. Object working memory relies heavily on attentional resources and visual encoding strategies during processing, which may render it vulnerable to sleep deprivation, particularly during information retention and updating (Shao et al., 2023). Extant direct comparative studies that examine the effects of caffeine and modafinil on the attenuation of sleep deprivation–induced impairments in object working memory are limited, particularly concerning their mechanisms of action during information encoding, maintenance, and retrieval. Therefore, emphasizing the need to further examine the stage-specific effects and neurophysiological basis of these two substances.

Event-related potential (ERP) technology is a widely utilized tool in cognitive neuroscience that enables real-time recording of the brain’s electrophysiological activity during cognitive processing. When studying working memory, the P2, N2, P3, and LPC components are critical ERP markers that are strongly associated with processes such as sensory processing, attentional control, information retention and updating, and cognitive control (Shao et al., 2024). The frontal P2 component is associated with early attentional allocation and feature evaluation during working memory processing (Potts, 2004), whereas the N2 and P3 waves, originating from the frontal region, represent top-down cognitive control and cognitive processing (Peng et al., 2020; Salmi et al., 2019). The LPC component, similar to the P3 component, reflects the attentional and orienting processes, particularly during the more refined stages of cognitive processing and evaluation (Shi et al., 2022). Given that both caffeine and modafinil exhibit cognitive-enhancing effects, this study utilized ERP technology to compare the differential patterns and neurophysiological mechanisms through which these substances mitigate impairments in object working memory after 36 h of total sleep deprivation. This study aimed to offer comprehensive evidence to elucidate cognitive enhancers’ effect on working memory in the context of sleep deprivation.

Caffeine predominantly enhances alertness, wakefulness, and response speed by antagonizing adenosine receptors, thereby nonspecifically inhibiting sleep pressure signaling. This effect is particularly pronounced during the early stages of working memory processing, such as perception and attentional control. Conversely, modafinil has a complex action mechanism, involving the modulation of the dopamine and norepinephrine systems, and predominantly enhances top-down cognitive control functions that are mediated by the prefrontal cortex. Therefore, this study hypothesized that the caffeine group would exhibit greater improvements in response speed, whereas the modafinil group would exhibit greater enhancement in task accuracy. Moreover, both substances are expected to improve performance by modulating different ERP components over time, reflecting the phase-specific nature of their action mechanisms, particularly during the encoding, maintenance, and retrieval stages of working memory. However, caffeine may exert a more substantial impact on early perceptual processing and attentional control (P2 and N2), whereas modafinil may have more significant effects during later stages of cognitive processing and control (P3 and LPC).

## 2 Materials and methods

### 2.1 Participants

Fourteen healthy male participants were recruited for this study (age range: 20–27 years; mean age: 23.5 years). All participants met the following inclusion criteria: right-handedness, normal or corrected vision, and no history of physical, psychiatric, or sleep disorders. The participants were assessed using face-to-face interviews and a self-developed sleep habit screening questionnaire. All participants reported the absence of difficulty falling asleep, circadian rhythm disorders, or excessive daytime sleepiness, and none of them had a recent history of drug use (including prescription medications, dietary supplements, and psychoactive substances). Participants were excluded if they regularly consumed caffeine, nicotine, alcohol, or tea.

Cognitive function screening indicated that participants had above-average memory quotients (MQ ≥ 110 on the Clinical Memory Scale) and Raven’s Progressive Matrices scores (IQ > 110). Additionally, the SCL-90 (Symptom Checklist-90) self-assessment scale scores were within the normal clinical range. Participants were required to maintain a regular sleep schedule of 7–8 h per night and refrain from consuming any beverages or medications containing caffeine or other psychoactive substances for one week before the experiment. This study was approved by the Beijing University of Aeronautics and Astronautics Ethics Committee. All participants were informed about the study procedures, sleep deprivation protocols, and potential risks, and provided written informed consent before participating in the experiment. The participants received compensation upon completion of the study.

### 2.2 Experimental task

**Figure 1:**
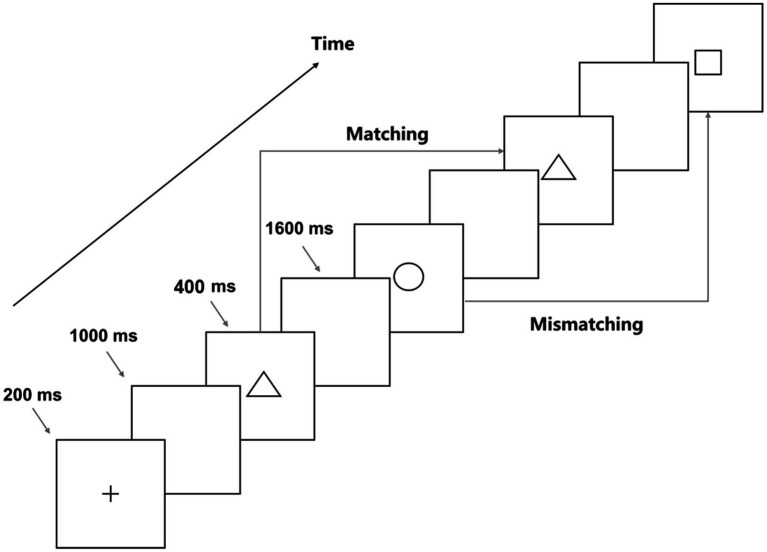
Schematic diagram of the object working memory task procedure.

This study employed a 2-back object working memory task to assess participants’ object working memory function. The task stimuli comprised 12 distinct geometric shapes, each presented with a size of 2° × 2°. The entire task comprised 122 trials with a completion time of approximately 5 min. Each trial began with the presentation of a white fixation cross (“+”) at the center of the screen for 200 ms. After the fixation cross disappeared, a blank screen was displayed for 1 s, followed by the presentation of the target geometric shape in the center of the screen for 400 ms. After the stimulus disappeared, an interval phase was presented, which lasted 1,600 ms. Participants were required to determine whether the current shape matched the shape presented two positions earlier (the task procedure is illustrated in Figure 1). If the shape matched, the participants pressed the left mouse button with their right index finger. If it did not match, they pressed the right mouse button with their right middle finger. The ratio of matching to non-matching trials was 1:1, and the trials were presented in random order. All participants received task training before the formal experiment to ensure that they fully understood the operational requirements. Only participants who achieved an accuracy rate >80% during the practice phase were permitted to proceed with the formal testing.

### 2.3 Experimental procedure

This study employed a randomized double-blind crossover design. All eligible participants were required to thoroughly understand the study procedure and complete the practice task before participating in the formal experiment. The experiment comprised two treatment conditions: caffeine and modafinil. Each participant completed two experimental sessions, spaced 3 weeks apart, to ensure adequate drug washout and recovery of cognitive function. The treatment’s order was randomly balanced across all participants to mitigate potential sequence effects.

Each experimental session lasted 3 days. Participants arrived at the laboratory at 18:00 (6 p.m.) on Day 1, where they spent the night with overnight sleep monitoring to ensure adequate rest. On Day 2 at 08:00 (8 a.m.), baseline EEG data during the object working memory task were collected. Following this, a period of 36 h of total sleep deprivation (TSD) was initiated. During the sleep deprivation period, participants were required to remain awake under continuous supervision by the research staff, avoid vigorous activities, and abstain from consuming any food or beverages containing stimulants or excitatory ingredients.

**Figure 2:**
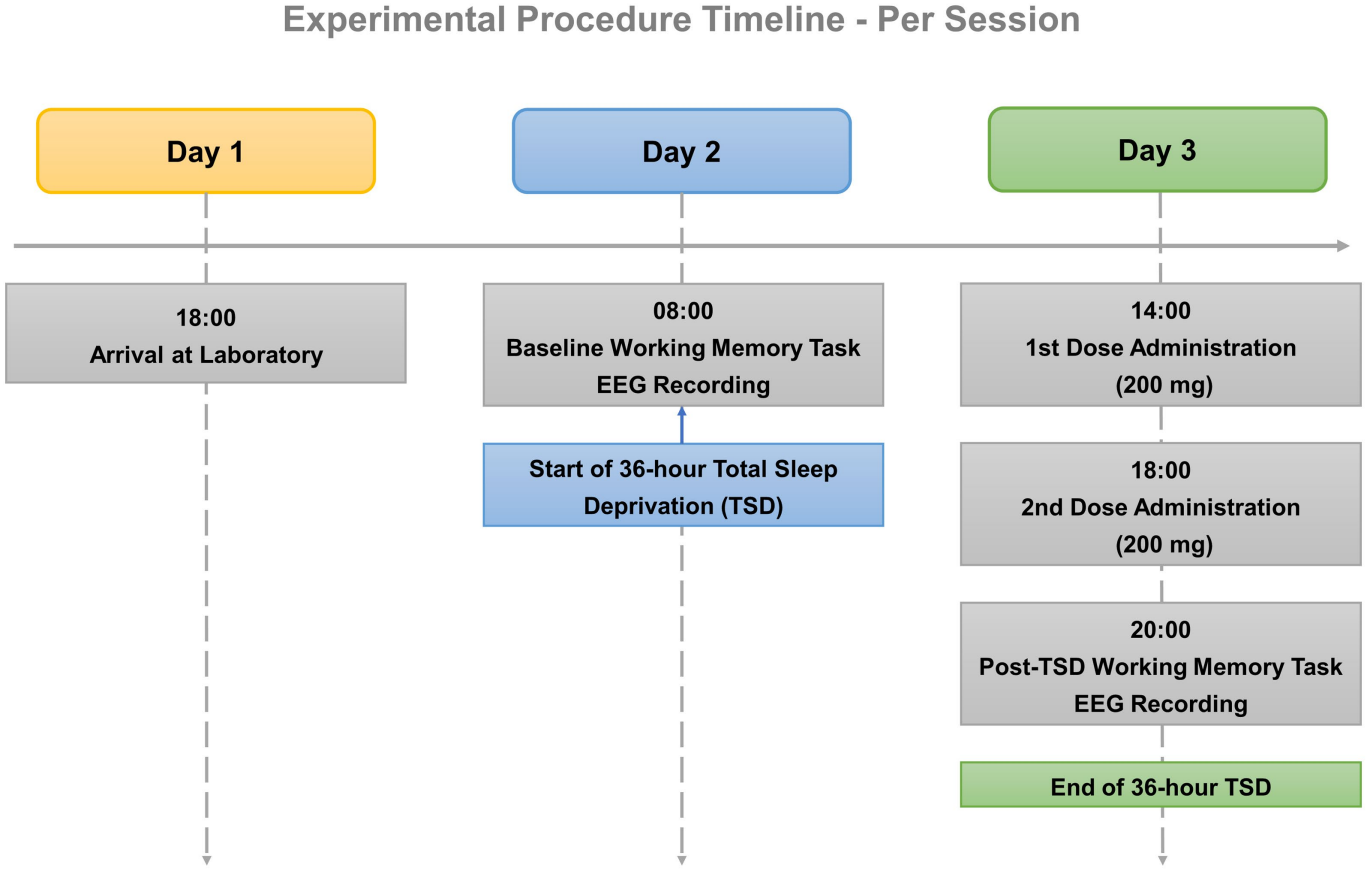
Timeline of the experimental procedure. Each participant completed two 3-day sessions separated by a three-week washout period. The 36-h total sleep deprivation (TSD) started immediately after the baseline task on Day 2 (08:00) and ended after the post-TSD task on Day 3 (20:00). Cognitive enhancers (caffeine/modafinil) were administered in two doses during the latter phase of the TSD.

On Day 3, at 14:00 (2 p.m.) and 18:00 (6 p.m.), participants received oral administration in two doses of 200 mg each, for a total cumulative dose of 400 mg. Both caffeine and modafinil were ground and encapsulated in identical gelatin capsules to achieve double blinding. The selection of a total dose of 400 mg was based on safety considerations established in previous research involving healthy adults (Sousa and Dinis-Oliveira, 2020; Verster and Koenig, 2018; Wesensten et al., 2004). A 4-h dosing interval was implemented, primarily referencing the 4–6-h half-life of caffeine, to ensure that drug concentrations remained stable throughout the testing period (Killgore et al., 2008). Subsequently, at 20:00 (8 p.m.) on Day 3 (exactly 36 h after the initiation of TSD), the post-deprivation EEG data during the object working memory task were collected. To provide a clearer overview of the timeline for task administration, sleep deprivation, and drug intervention, a schematic diagram of the experimental protocol is presented in Figure 2.

To ensure compliance with the sleep deprivation protocol and to monitor participants’ wakefulness throughout the procedure, two trained research staff members accompanied each participant continuously for the entire 36-h sleep deprivation period. The laboratory environment was strictly controlled to eliminate any conditions conducive to sleep onset. During this period, participants were permitted to engage in light recreational activities, such as reading or watching low-intensity films, but were prohibited from performing vigorous physical exercise or consuming caffeinated beverages. All meals were standardized and provided by the research team to minimize uncontrolled dietary influences.

### 2.4 EEG data acquisition and recording

The EEG signals were recorded using a 32-channel NeuroScan system (Compumedics, Charlotte, NC, USA). All electrode impedances were maintained below 5 kΩ, with a sampling rate of 1,000 Hz. Data processing was conducted in MATLAB 2018b using the EEGLAB toolbox (Delorme and Makeig, 2004). The raw data were subjected to band-pass filtering between 0.1 and 30 Hz to eliminate low-frequency drift and high-frequency noise. Data segments were extracted based on the onset of memory stimuli, ranging between −200 ms and 800 ms, with −200 ms, preceding the stimulus presentation, used as the baseline for correction. Prior to independent component analysis (ICA) decomposition, epochs containing excessive drifts or large-amplitude artifacts were removed, reducing the dataset from 6,344 to 6,130 trials. ICA was applied to decompose EEG signals into statistically independent components. Artifact-related components were identified using ICLabel (Pion-Tonachini et al., 2019) and automatically flagged for removal if classified as non-brain artifacts (Muscle, Eye, Heart, Line Noise, or Channel Noise) with a probability exceeding 0.70. All flagged components were subsequently verified through manual visual inspection of scalp topographies and component time courses prior to removal. On average, 2.192 ± 1.127 ICs were removed across all conditions, the majority of which were ocular artifacts (69.298%). Following artifact component removal, EEG data were re-referenced to the average of all scalp electrodes. Subsequently, ICA-identified artifact components were removed, and trials exceeding ±100 μV were automatically excluded to maintain data integrity, resulting in the rejection of an additional 27 trials (0.44%). The final dataset retained 6,103 trials across all conditions. The mean (±SD) numbers of correct trials retained per condition were as follows: Modafinil-Baseline: 108.46 ± 4.94; Modafinil-TSD: 108.69 ± 4.27; Caffeine-Baseline: 108.23 ± 4.87; Caffeine-TSD: 104.54 ± 13.97.

### 2.5 Data analysis

#### 2.5.1 Behavioral data analysis

Reaction time (RT) and accuracy were used to assess working memory performance across experimental conditions. All data are expressed as mean ± standard deviation. A 2 (drug: caffeine vs. modafinil) × 2 (sleep state: baseline vs. 36-h total sleep deprivation) two-way repeated-measures analysis of variance (ANOVA) was conducted separately for RT and accuracy. Where a significant interaction was observed, simple effects analyses were performed to decompose the interaction by examining the effect of each factor at individual levels of the other factor. Post-hoc pairwise comparisons were corrected using Tukey’s Honestly Significant Difference (HSD) test, which controls the familywise error rate while maintaining high statistical power for pairwise comparisons.

#### 2.5.2 ERP data analysis

ERP data analysis was performed on all correct trials. Four ERP components were analyzed in the present study: P2, N2, P3, and LPC. Epochs were time-locked to the onset of the memory probe stimulus, spanning from 200 ms pre-stimulus to 800 ms post-stimulus, with the200 ms pre-stimulus interval serving as the baseline for amplitude correction. For ERP component quantification, an *a priori* frontal region of interest (ROI) comprising electrodes F3, Fz, and F4 was selected based on the theoretical focus of the study. Given that the 2-back task heavily recruits the prefrontal cortex for working memory maintenance and top-down cognitive control, and that modafinil’s cognitive-enhancing mechanism is primarily linked to catecholaminergic modulation within the prefrontal cortex, this frontal ROI was adopted to systematically track neural dynamics at modafinil’s primary cortical target across all processing stages within a single, theoretically coherent framework (Gevins and Smith, 2000).

The component-specific rationale for frontal quantification is as follows. The P2 (100–200 ms) at frontal sites reflects early attentional allocation processes mediated by the prefrontal cortex. The N2 (200–300 ms) is well-documented at frontal and frontocentral sites in the context of cognitive control and response conflict monitoring, reflecting the engagement of the anterior cingulate cortex and lateral prefrontal cortex in inhibitory control and conflict resolution (Folstein and Van Petten, 2008). Although the classic P3b is typically maximal at parietal sites in simple oddball paradigms, a substantial body of literature demonstrates that n-back working memory tasks and sleep deprivation conditions consistently produce a frontally dominant P3 distribution, reflecting heightened recruitment of prefrontal executive resources during working memory updating and maintenance (Polich, 2007; Gevins and Smith, 2000; Gosselin et al., 2005). The LPC (450–800 ms), reflecting sustained attentional processing and post-decisional memory encoding, has similarly been reported at frontal sites under conditions of elevated cognitive load and working memory demand (Kok, 2001; Gevins and Smith, 2000).

For P2, N2, and P3, the peak amplitude (maximum positive deflection for P2 and P3; maximum negative deflection for N2) was extracted within the predefined time window at each of the three electrodes individually. For the LPC, given its characteristically broad and sustained deflection, the mean amplitude across the 450–800 ms window was extracted at each electrode. In all cases, the resulting values were then averaged across F3, Fz, and F4 to yield a single amplitude measure per participant per condition. The predefined time windows were as follows: P2 (100–200 ms), N2 (200–300 ms), P3 (300–450 ms), and LPC (450–800 ms), all defined based on the grand-average waveforms and established ERP literature.

A 2 (drug: caffeine vs. modafinil) × 2 (sleep state: baseline vs. 36-h total sleep deprivation) two-way repeated-measures ANOVA was conducted for each component amplitude. Where a significant interaction was observed, simple effects analyses were performed, with post-hoc pairwise comparisons corrected using Tukey’s HSD test, which controls the familywise error rate while maintaining high statistical power for pairwise comparisons.

## 3 Results

### 3.1 Behavioral results

#### 3.1.1 Reaction time

**Figure 3:**
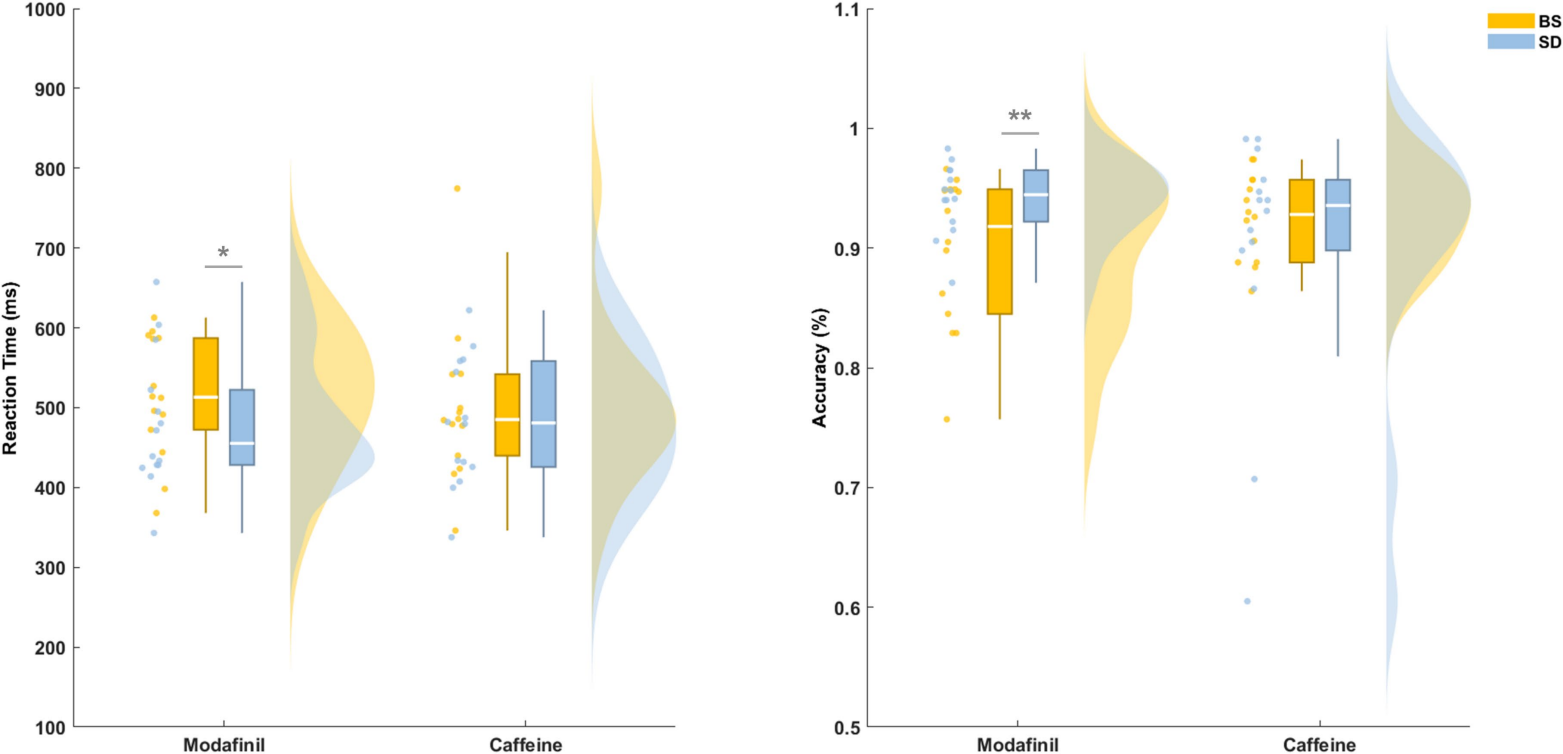
Statistical visualization of object working memory reaction time and accuracy in the modafinil and caffeine groups before and after sleep deprivation. BS: Baseline sleep; SD: sleep deprivation. Yellow: BS condition; Blue: SD condition. *p < 0.05, **p < 0.01.

The main effect of sleep condition was not significant (*F*_(1, 13)_ = 3.077, *p* = 0.103, *η_p_*^2^ = 0.191), nor was the main effect of the cognitive enhancer (*F*_(1, 13)_ = 0.227, *p* = 0.642, *η_p_*^2^ = 0.017). Additionally, no significant interaction was found between sleep condition and cognitive enhancer (*F*_(1, 13)_ = 0.336, *p* = 0.572, *η_p_*^2^ = 0.025; Table 1 and Figure 3).

**Table 1:** Descriptive statistics of object working memory reaction time and accuracy in the modafinil and caffeine groups before and after 36 h of sleep deprivation (M ± SD).

Cognitive enhancer	Sleep state	RT (ms)	Accuracy (%)
Modafinil	BS	514.010 ± 76.081	89.800 ± 6.376
	36 h-TSD	480.437 ± 85.645	94.115 ± 2.979
Caffeine	BS	499.505 ± 99.204	92.569 ± 3.527
	36 h-TSD	481.981 ± 81.192	89.831 ± 11.049

#### 3.1.2 Accuracy

The main effect of sleep condition was not significant (*F*_(1, 13)_ = 0.257, *p* = 0.621, *η_p_*^2^ = 0.019), and the main effect of the cognitive enhancer was not significant (*F*_(1, 13)_ = 0.272, *p* = 0.611, *η_p_*^2^ = 0.020). However, the interaction between sleep condition and cognitive enhancer was significant (*F*_(1, 13)_ = 6.356, *p* = 0.026, *η_p_*^2^ = 0.328). Furthermore, object working memory accuracy significantly increased after 36 h of sleep deprivation following modafinil administration (*p* = 0.009), whereas no significant difference in accuracy was found before and after sleep deprivation when caffeine was administered (*p* = 0.313; Table 1 and Figure 3).

### 3.2 ERP results

#### 3.2.1 P2 component

**Figure 4:**
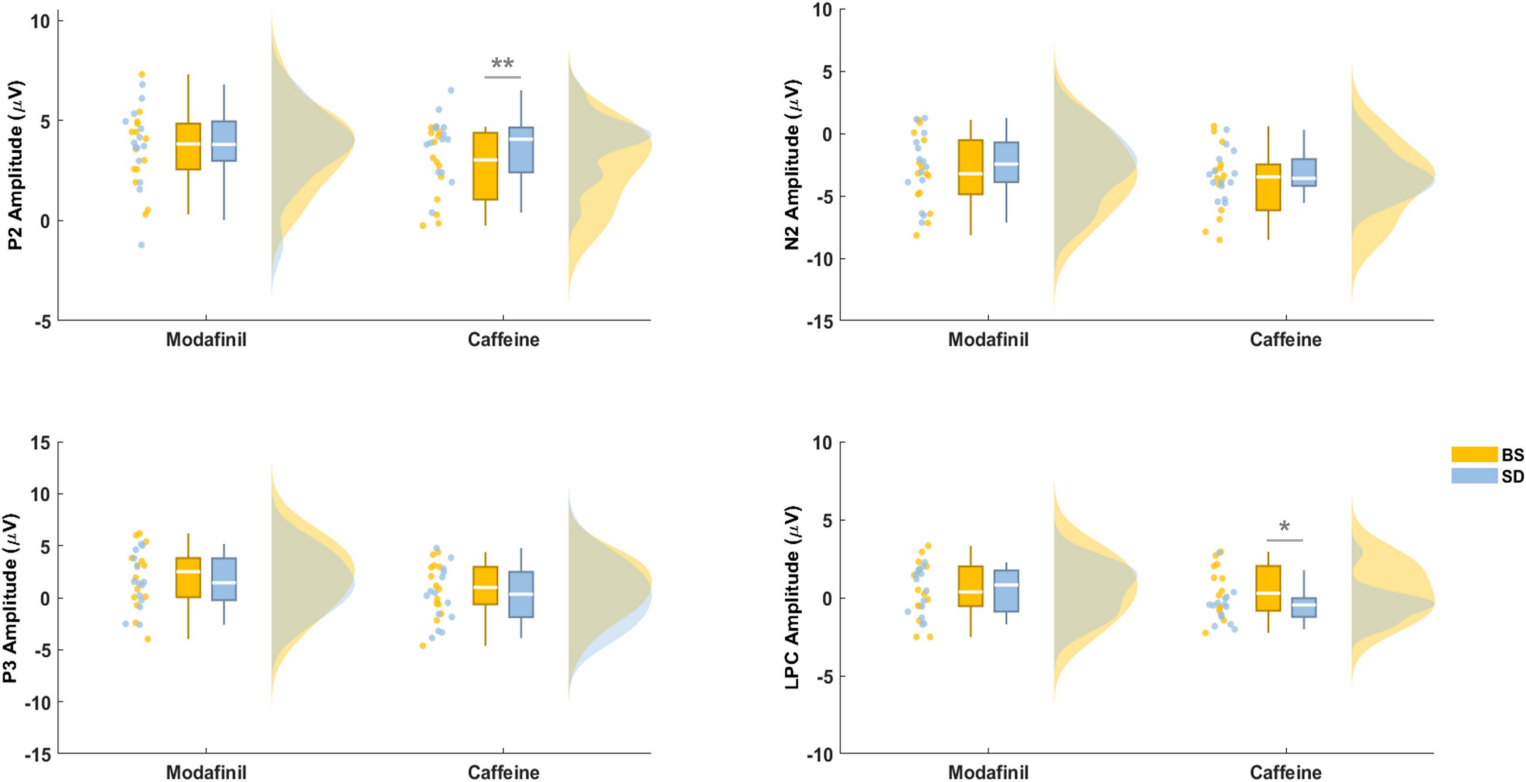
Amplitude of ERP components (P2, N2, P3, and LPC) under baseline (BS) and sleep deprivation (SD) conditions for the modafinil and caffeine groups. BS: Baseline sleep; SD: Sleep deprivation. Yellow: BS condition; Blue: SD condition. *p < 0.05, **p < 0.01.

**Figure 5:**
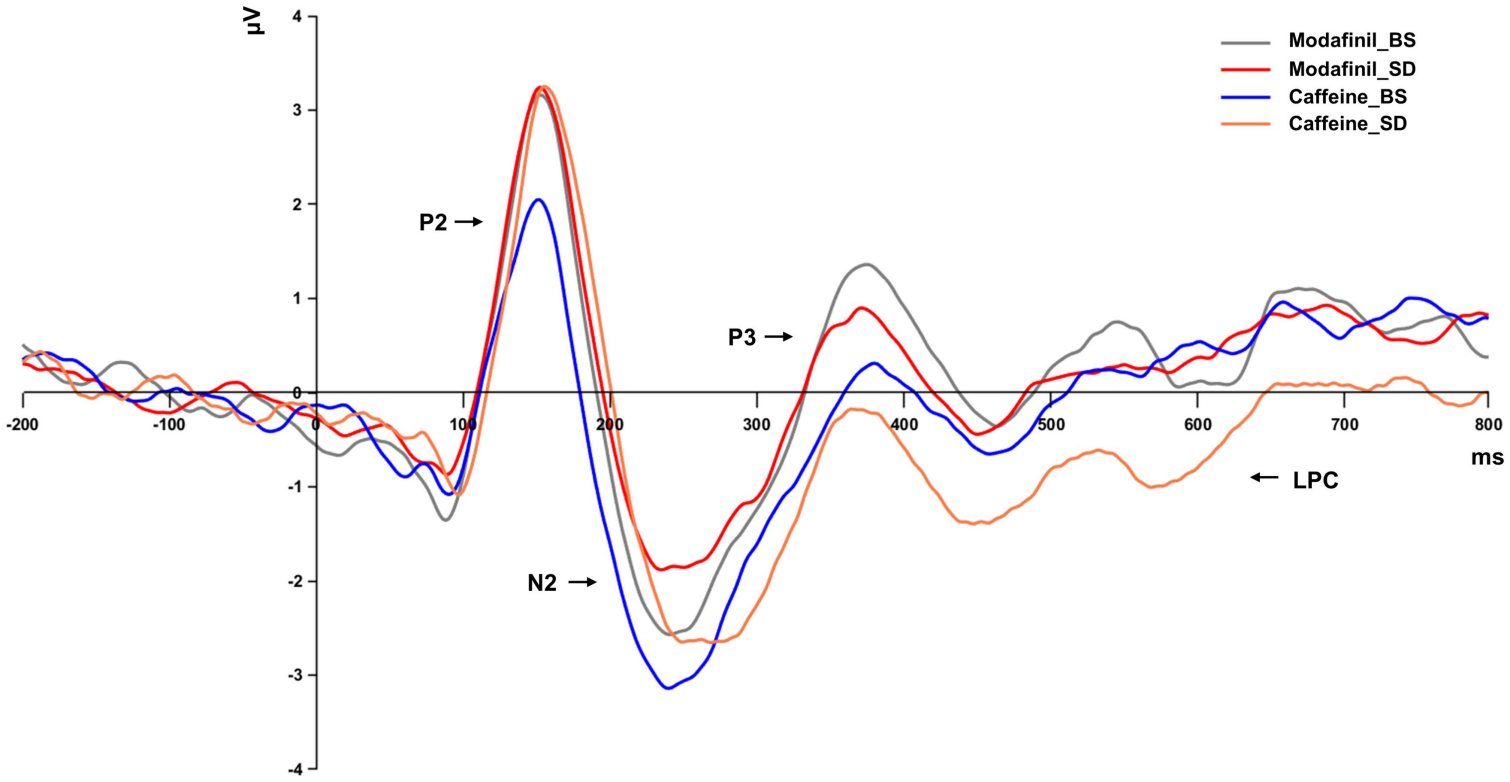
ERP waveforms of the caffeine and modafinil groups before and after sleep deprivation.

**Figure 6:**
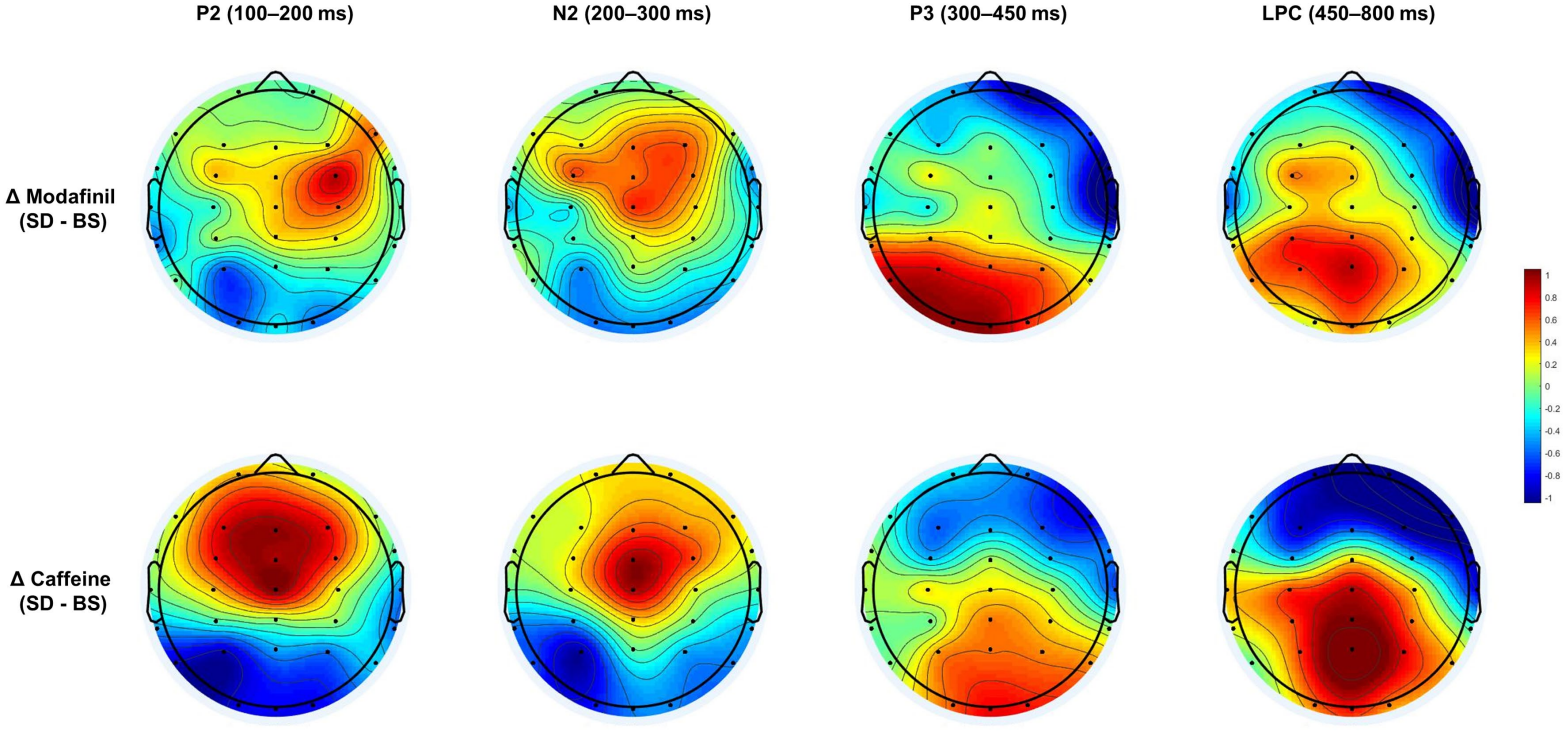
Topographical maps of difference waves (SD - Baseline) for ERP components. The scalp maps display the grand-average amplitude differences (*Δ*) for P2 (100–200 ms), N2 (200–300 ms), P3 (300–450 ms), and LPC (450–800 ms) under modafinil and caffeine conditions.

The interaction between sleep condition and cognitive enhancer was significant (*F*_(1, 13)_ = 5.579, *p* = 0.034, *η_p_*^2^ = 0.300). The simple effects analysis revealed no significant difference after modafinil administration in the P2 amplitude before and after 36 h of sleep deprivation (*p* = 0.747). However, regarding the caffeine group, the P2 amplitude significantly increased after 36 h of sleep deprivation compared with the baseline (*p* = 0.004). The main effect of sleep condition was not significant (*F*_(1, 13)_ = 3.162, *p* = 0.099, *η_p_*^2^ = 0.196), and the main effect of the cognitive enhancer was not significant (*F*_(1, 13)_ = 1.656, *p* = 0.221, *η_p_*^2^ = 0.113; Table 2 and Figures 4–6).

**Table 2:** Descriptive statistics of ERP component amplitudes in the modafinil and caffeine groups before and after 36 h of sleep deprivation (M ± SD).

Cognitive enhancer	Sleep state	P2 (*μ*V)	N2 (*μ*V)	P3 (*μ*V)	LPC (*μ*V)
Modafinil	BS	3.567 ± 1.914	−3.205 ± 2.903	1.918 ± 3.093	0.494 ± 1.895
	36 h-TSD	3.722 ± 2.023	−2.670 ± 2.710	1.509 ± 2.574	0.444 ± 1.452
Caffeine	BS	2.733 ± 1.822	−3.738 ± 2.810	0.849 ± 2.610	0.422 ± 1.655
	36 h-TSD	3.806 ± 1.568	−3.278 ± 1.753	0.202 ± 2.746	−0.490 ± 1.214

#### 3.2.2 N2 component

The interaction between sleep condition and cognitive enhancer was not significant (*F*_(1, 13)_ = 0.024, *p* = 0.879, *η_p_*^2^ = 0.002). The main effect of sleep condition was not significant (*F*_(1, 13)_ = 2.372, *p* = 0.148, **η* *_p_* ^2^* = 0.154), and the main effect of the cognitive enhancer was not significant (*F*_(1, 13)_ = 2.785, *p* = 0.119, **η* *_p_* ^2^* = 0.176; Table 2 and Figures 4–6).

#### 3.2.3 P3 component

The interaction between sleep condition and cognitive enhancer was not significant (*F*_(1, 13)_ = 0.198, *p* = 0.664, *η_p_*^2^ = 0.015). The main effect of sleep condition was not significant (*F*_(1, 13)_ = 2.095, *p* = 0.171, *η_p_*^2^ = 0.139); however, the main effect of the cognitive enhancer was significant (*F*_(1, 13)_ = 10.383, *p* = 0.007, *η_p_*^2^ = 0.444). Given that the baseline measurements were taken prior to drug administration and the interaction was not significant, this main effect of the session cannot be attributed to the pharmacological intervention. It may reflects a pre-existing inter-session baseline difference that remained unchanged across the combination of sleep deprivation and drug administration (Table 2 and Figures 4–6).

#### 3.2.4 LPC component

The interaction between sleep condition and cognitive enhancer was significant (*F*_(1, 13)_ = 5.606, *p* = 0.034, *η_p_*^2^ = 0.301). The simple effects analysis revealed no significant difference in the LPC amplitude after modafinil administration before and after 36 h of sleep deprivation (*p* = 0.878). Conversely, the LPC amplitude significantly reduced in the caffeine group after 36 h of sleep deprivation compared with baseline (*p* = 0.047). The main effect of sleep condition was not significant (*F*_(1, 13)_ = 2.217, *p* = 0.160, *η_p_*^2^ = 0.146), and the main effect of the cognitive enhancer was not significant (*F*_(1, 13)_ = 3.093, *p* = 0.102, *η_p_*^2^ = 0.192; Table 2 and Figures 4–6).

To further address potential inter-session baseline variability inherent to the crossover design, a supplementary difference-score analysis was conducted. For each participant and each ERP component, a change score (*Δ* = post-TSD amplitude − baseline amplitude) was calculated under each drug condition, and paired-samples t-tests were used to compare the Δ scores between caffeine and modafinil. The results confirmed that caffeine produced a significantly larger increase in P2 amplitude relative to modafinil (*t*_(13)_ = −2.362, *p* = 0.034), and a significantly larger reduction in LPC amplitude relative to modafinil (*t*_(13)_ = 2.368, *p* = 0.034). No significant between-drug differences in Δ scores were observed for N2 (*t*_(13)_ = 0.155, *p* = 0.879) or P3 (*t*_(13)_ = 0.445, *p* = 0.664). These findings are fully consistent with the primary ANOVA results, reinforcing the conclusion that caffeine and modafinil produce distinct patterns of ERP change following sleep deprivation.

## 4 Discussion

This study compared the counteractive effects of modafinil and caffeine on object working memory after 36 h of complete sleep deprivation. The results suggested that both cognitive enhancers can mitigate sleep deprivation-induced cognitive decline to some extent; however, their action mechanisms differ. Behaviorally, modafinil not only counteracted the performance impairments associated with sleep deprivation but also reduced reaction times and improved accuracy, demonstrating performance enhancement beyond baseline levels. Conversely, caffeine did not produce significant behavioral improvements across sleep deprivation conditions and appeared to predominantly maintain baseline performance. The ERP metrics clarified the distinct neural processing pathways associated with each compound. Modafinil was linked to stable P2 and LPC components across deprivation states, suggesting that its efficacy may stem from improved early neural efficiency and sustained top-down cognitive processing. Conversely, caffeine significantly increased P2 and LPC amplitudes after sleep deprivation, indicating a reliance on augmented early perceptual and attentional processing as well as compensatory neural mechanisms to maintain task performance under conditions of fatigue. Therefore, modafinil resulted in a more direct and efficient cognitive enhancement profile, whereas caffeine relied heavily on compensatory neural resources to offset the adverse effects of sleep deprivation.

From a behavioral perspective, the results of this study provide evidence for differential effects of modafinil and caffeine in mitigating object working memory impairments caused by 36 h of complete sleep deprivation. The primary confirmatory behavioral finding is the significant Drug × Sleep State interaction in accuracy, which demonstrates that modafinil significantly improved task accuracy following sleep deprivation, whereas caffeine did not produce a significant change. This interaction indicates that modafinil produced a genuine enhancement in working memory precision that surpassed baseline performance, while caffeine appeared to maintain accuracy at baseline levels, thereby preventing the cognitive decline typically associated with prolonged sleep deprivation (Killgore et al., 2009; Wesensten et al., 2002).

This dissociation in behavioral outcomes may stem from inherent pharmacological and cognitive mechanisms that differentiate between the two cognitive enhancers. As a nonselective adenosine receptor antagonist, caffeine predominantly increases global cortical arousal by blocking sleep pressure signaling (O'Callaghan et al., 2018), which can transiently enhance alertness and perceptual responses, thereby preventing further cognitive performance degradation. However, owing to the lack of selective regulation over specific cognitive processes, its enhancement effects may be limited to basic perceptual arousal and attention maintenance, rendering it less effective in robustly improving information-processing efficiency during high-load working memory tasks (McLellan et al., 2016). Conversely, modafinil enhanced top-down cognitive control and sustained attention, particularly in the prefrontal cortex, by modulating the catecholamine system, most notably by increasing dopamine and norepinephrine concentrations in the synaptic cleft (Punzi et al., 2017). This allows the brain to efficiently encode and update information, even under conditions of extreme fatigue (Battleday and Brem, 2015). This mechanism not only contributes to the dual enhancement observed in response times and accuracy with modafinil but is also highly consistent with previous studies reporting the efficacy of modafinil in enhancing executive functions and working memory performance (Gill et al., 2006).

The behavioral dissociation observed in this study suggests that these two drugs may influence different stages of cognitive processing. Given that working memory involves a multistage sequence (from early perception to subsequent updating), ERP technology offers valuable insights into stage-specific neural dynamics. Furthermore, this study examined ERP components (P2, N2, P3, and LPC) to determine potential dissociations in the neural mechanisms underlying the information encoding, control, and updating of the two substances.

The P2 component reflects early attentional allocation and feature evaluation and captures the brain’s initial response to external stimuli. These results suggest that caffeine significantly increases the P2 amplitude following 36 h of sleep deprivation, indicating its role in enhancing early perceptual processing and visual attention. Caffeine non-specifically increases cortical excitability by antagonizing adenosine receptors, thereby elevating alertness and mobilizing additional neural resources during the early stages of information processing to counteract the heightened sensory thresholds induced by fatigue. This phenomenon may be interpreted as a “compensatory hyperarousal mechanism,” in which caffeine assists in maintaining sensitivity to environmental stimuli by reinforcing attentional resource deployment during the early perceptual phase after sleep deprivation (Chen et al., 2020). Conversely, modafinil elicited no significant changes in P2 amplitude, which suggests that its primary effects are exerted during the later stages of cognitive control and higher-level processing rather than through compensatory hyperactivation of early perceptual pathways (Battleday and Brem, 2015). Previous modern neuroimaging and electrophysiological studies support this interpretation, indicating that modafinil predominantly enhances functional connectivity and neural efficiency in the prefrontal cortex by modulating the catecholaminergic system. This modulation selectively improves higher-order executive functions that depend on top-down control, rather than simply amplifying early sensory input (Punzi et al., 2017; Saletu et al., 2009).

The N2 component is predominantly associated with conflict detection, decision making, and early cognitive control (Folstein and Van Petten, 2008). In this study, the N2 amplitude exhibited no statistically significant variation, indicating that modafinil and caffeine may exert similar effects on conflict monitoring and decision-making control processes during sleep deprivation. This similarity may arise from a shared capacity to enhance top-down cognitive control mechanisms, thereby mitigating sleep deprivation’s adverse effects. The absence of statistically significant differences in the N2 component may be attributable to the relatively low demand for conflict detection at this early processing stage, as well as the possibility that the two drugs modulate this function via distinct neural pathways; however, their effects may converge functionally at this stage (Ray et al., 2012).

The P3 component is typically associated with higher-order cognitive processing and working memory regulation (Polich, 2007). In this study, P3 amplitude was consistently higher under modafinil than under caffeine, suggesting a tonic, session-wide facilitation of frontal cognitive resources rather than a selective post-deprivation effect. This sustained effect likely reflects modafinil’s continuous promotion of prefrontal executive control (Minzenberg and Carter, 2008; Volkow et al., 2009). While caffeine supports the processing of early perceptual inputs, modafinil appears to facilitate general encoding and executive functions providing a stable cognitive baseline regardless of sleep state.

The LPC component reflects sustained attentional processing and late-stage cognitive maintenance during working memory tasks (Citron, 2012). In the present study, the caffeine group showed a significant decrease in LPC amplitude following 36 h of sleep deprivation, whereas the modafinil group maintained stable LPC amplitude. This dissociation suggests that, despite caffeine’s ability to enhance early perceptual processing (as reflected by increased P2 amplitude), it failed to preserve the neural resources required for late-stage information maintenance under conditions of extreme fatigue. The reduction in LPC amplitude in the caffeine group may indicate progressive depletion of higher-order cognitive maintenance capacity, consistent with caffeine’s primary mechanism of non-selective adenosine receptor antagonism, which broadly elevates arousal but does not selectively support prefrontal-dependent maintenance processes (McLellan et al., 2016). In contrast, the stability of LPC amplitude in the modafinil group suggests that modafinil’s catecholaminergic modulation of the prefrontal cortex sustains late-stage cognitive maintenance more effectively. Together, these findings reinforce the conclusion that modafinil provides more comprehensive and efficient neuroprotection across the full temporal cascade of working memory processing.

Although this study revealed the differing neurocognitive modulation mechanisms of modafinil and caffeine under sleep deprivation conditions, several limitations must be considered when interpreting the results. First, the sample size was relatively small. Although a within-subject repeated-measures design was employed, which mitigated individual differences to some extent and enhanced statistical power, the limited sample size may have constrained the statistical analyses’ sensitivity. Future studies should aim to increase the sample size to validate the robustness of this study’s findings. Second, to exclude the potential confounding effects of hormonal fluctuations related to the female physiological cycle on sleep physiology and drug metabolism, this study only included male participants. Consequently, the conclusions drawn may not be directly applicable to the female population. Given that gender differences may play a role in drug responses and anti-fatigue mechanisms, future studies should explore this issue in gender-balanced samples. Third, this study utilized a single fixed dose (200 mg of modafinil and caffeine). Owing to the absence of a multi-dose gradient design, this study was unable to examine the dose-effect relationships of these two cognitive enhancers or determine whether an “optimal dose” existed, which may have yielded superior cognitive benefits. Although the selected doses were commonly used as functional doses in previous studies, individual differences in drug tolerance and metabolic rates may not have been fully accounted for. Fourth, this study did not include a placebo group. Although this study aimed to directly compare the differences between modafinil and caffeine, the lack of a placebo group limited this study’s ability to accurately quantify the absolute net effects of both substances relative to a “pure sleep deprivation state,” and did not completely rule out the potential contributions of practice effects or placebo effects on behavioral outcomes. However, the significant and specific differences observed in the ERP components between the two substances strongly support the notion that they operate through distinct neurobiological mechanisms, rather than being solely attributable to placebo effects. (5) This session-to-session variability in baseline levels is an inherent constraint of longitudinal crossover paradigms and should be considered when interpreting condition differences observed in the present study. To mitigate this issue in future research, we recommend incorporating pre-session state verification procedures at the beginning of each experimental session. Specifically, administering the Psychomotor Vigilance Task prior to the main cognitive task would provide an objective, standardized index of vigilance and alertness, enabling researchers to confirm that participants’ arousal states are comparable across sessions. Complementing this with brief subjective sleepiness scales would further characterize participants’ subjective fatigue states. Additionally, recording a short segment of resting-state EEG at the outset of each session would yield an objective neurophysiological baseline against which task-related activity could be normalized. Together, these measures would allow researchers to statistically verify baseline equivalence across sessions and, where necessary, include pre-session state indices as covariates in subsequent analyses, thereby substantially reducing the confounding influence of inter-session variability in future longitudinal crossover designs. (6) Due to the relatively small sample size, ERP responses in the N-back task were analyzed across all trials, rather than separately for target and non-target stimuli. While averaging across all trials is a common practice in the N-back ERP literature, this approach may overlook potential differences in neural processing between targets and non-targets. Future studies with larger sample sizes could investigate target- and non-target-specific ERP responses and their differential sensitivity to sleep deprivation and cognitive enhancers.

This study integrated behavioral analysis with high-temporal-resolution ERP techniques to systematically examine the differential neurocognitive mechanisms through which modafinil and caffeine sustain object working memory during extreme sleep deprivation (36 h). The findings revealed distinct anti-fatigue neurostrategies associated with the two cognitive enhancers, characterized by specific spatiotemporal dissociations. The core findings suggested that, although both substances effectively mitigate sleep deprivation-induced cognitive decline, their underlying neurocognitive mechanisms differ fundamentally.

Caffeine primarily induces a compensatory hyperarousal mode that enhances early perceptual processing, but fails to sustain late-stage cognitive maintenance resources, suggesting that its compensatory capacity is limited to earlier stages of information processing. This strategy compensates for fatigue-related declines in processing efficiency, albeit at the cost of increased neural expenditure, thereby preserving behavioral performance at baseline levels. Conversely, modafinil exhibited superior neural efficiency, selectively enhancing resource availability during the core stages of cognitive evaluation and executive control while minimizing reliance on compensatory perceptual processing. This top-down precision regulation facilitated more efficient cognitive resource allocation and enabled participants to achieve confirmed performance gains in accuracy surpassing baseline levels, with a convergent exploratory trend in response speed.

This study provides direct electrophysiological evidence that differentiates bottom-up generalized arousal (mediated by adenosine antagonism) from top-down specific cognitive enhancement (regulated by catecholaminergic modulation). This mechanistic dissociation emphasizes the importance of aligning antifatigue strategies with task-specific cognitive demands. Caffeine is an effective intervention for extended tasks that require sustained alertness and basic perceptual processing. Conversely, modafinil provided superior and more efficient cognitive support in high-stakes tasks involving heavy working memory load, complex decision-making, and strict accuracy demands.

## Data Availability

The raw data supporting the conclusions of this article will be made available by the authors, without undue reservation.
